# Exploring the Time Trend of Stress Levels While Using the Crowdsensing Mobile Health Platform, TrackYourStress, and the Influence of Perceived Stress Reactivity: Ecological Momentary Assessment Pilot Study

**DOI:** 10.2196/13978

**Published:** 2019-10-30

**Authors:** Rüdiger Pryss, Dennis John, Winfried Schlee, Wolff Schlotz, Johannes Schobel, Robin Kraft, Myra Spiliopoulou, Berthold Langguth, Manfred Reichert, Teresa O'Rourke, Henning Peters, Christoph Pieh, Claas Lahmann, Thomas Probst

**Affiliations:** 1 Institute of Databases and Information Systems Ulm University Ulm Germany; 2 Lutheran University of Applied Sciences Nuremberg Germany; 3 Department of Psychiatry and Psychotherapy University of Regensburg at Bezirksklinikum Regensburg Germany; 4 Max Planck Institute for Empirical Aesthetics Frankfurt Germany; 5 Faculty of Computer Science Otto-von-Guericke-University Magdeburg Germany; 6 Department for Psychotherapy and Biopsychosocial Health Danube University Krems Krems Austria; 7 Department of Psychiatry and Psychotherapy LMU Munich Munich Germany; 8 Faculty of Medicine, Department of Psychosomatic Medicine and Psychotherapy Medical Center-University of Freiburg Freiburg Germany

**Keywords:** mHealth, psychological stress, crowdsensing, ecological momentary assessment, pilot study

## Abstract

**Background:**

The mobile phone app, TrackYourStress (TYS), is a new crowdsensing mobile health platform for ecological momentary assessments of perceived stress levels.

**Objective:**

In this pilot study, we aimed to investigate the time trend of stress levels while using TYS for the entire population being studied and whether the individuals’ perceived stress reactivity moderates stress level changes while using TYS.

**Methods:**

Using TYS, stress levels were measured repeatedly with the 4-item version of the Perceived Stress Scale (PSS-4), and perceived stress reactivity was measured once with the Perceived Stress Reactivity Scale (PSRS). A total of 78 nonclinical participants, who provided 1 PSRS assessment and at least 4 repeated PSS-4 measurements, were included in this pilot study. Linear multilevel models were used to analyze the time trend of stress levels and interactions with perceived stress reactivity.

**Results:**

Across the whole sample, stress levels did not change while using TYS (*P*=.83). Except for one subscale of the PSRS, interindividual differences in perceived stress reactivity did not influence the trajectories of stress levels. However, participants with higher scores on the PSRS subscale reactivity to failure showed a stronger increase of stress levels while using TYS than participants with lower scores (*P*=.04).

**Conclusions:**

TYS tracks the stress levels in daily life, and most of the results showed that stress levels do not change while using TYS. Controlled trials are necessary to evaluate whether it is specifically TYS or any other influence that worsens the stress levels of participants with higher reactivity to failure.

## Introduction

### Background

Selye introduced the term stress as the “nonspecific response of the body to any demand made upon it” [1]. According to the transactional model of stress by Lazarus and Folkman [2], 2 cognitive processes (primary and secondary appraisal) determine the individual experience of stress. Primary appraisal reflects an individual’s evaluation of the situation being relevant and potential threats, whereas secondary appraisal reflects an individual’s evaluation of manageability of the situation. If the situation is considered relevant and one’s own capacities are considered insufficient to deal with the situation, then stress is the consequence. Note that neuroscience showed that stress involves reactions in the central and peripheral nervous system [3].

Continuously elevated stress levels can increase the risk of mental and somatic disease [3,4]. Stress levels vary between and within individuals. Individuals differ in their stress levels because of their genetics, developmental experiences, and personality traits to provide only some examples [3]. An important concept in this context constitutes perceived stress reactivity, which has been defined as *a disposition that underlies relatively stable individual differences in stress responses*. [5]. Within an individual, stress levels can vary depending on, for instance, the severity, intensity, and the frequencies of stressors [3]. Therefore, the assessment of inter- and intraindividual aspects of stress levels is of paramount importance, for example, for reducing the risk to develop mental or somatic disorders.

### Objectives

Ecological momentary assessments (EMAs) of stress levels allow the investigation of these inter- and intraindividual differences under real-life conditions [6]. Although a recent review on mobile phone–based stress assessments included 35 studies [7], validated stress scales were used only in 5 of these studies. In this study, we present the *TrackYourStress* (TYS) crowdsensing mobile health (mHealth) platform that comprises the short version of the validated Perceived Stress Scale (PSS-4; [8]). Although the PSS-4 received some criticism [9,10], both the reliability and validity were acceptable in a European study [11]. We selected the PSS-4 instead of, for example, longer versions of the PSS [8] or the Perceived Stress Questionnaire (PSQ; [12]) for TYS, as scales for mobile phone–based assessments should be both psychometrically sound and as short as possible, so that participants are willing to fill in the scale continuously over a longer period of time and without (1) any bias because of measurement reactivity or (2) missing data because of missed signals. TYS combines EMAs and crowdsensing by solely using mobile technology and by integrating mobile phone sensors to collect data. In another study [13], we investigated passively sensed environmental data (Global Positioning System [GPS] location of TYS users) as predictors of daily measured stress-related items. In contrast to the previous study, this paper introduces TYS in more detail and reports results of an explorative pilot study on the trajectories of perceived stress levels assessed weekly with the PSS-4 in nonclinical TYS users to investigate measurement reactivity, that is, the extent to which measuring affects the measurement itself. The following 2 research questions (RQ) were addressed:

- RQ1 refers to average measurement reactivity in the total sample: How is the time trend of stress levels when using TYS in the entire population being studied in a first explorative analysis?
- RQ2 refers to interindividual differences in measurement reactivity: Is there an interaction between the time trend of stress levels when using TYS and interindividual differences in perceived stress reactivity? As perceived stress reactivity and stress levels have been shown to be correlated in the cross-sectional research [5], we explored whether the individuals’ perceived stress reactivity interacts not only with simultaneously measured stress levels but also with longitudinally measured stress trajectories.

## Methods

### Measures

#### Perceived Stress Scale

The PSS-4 [8] was used in this study to operationalize stress levels using repeated measures across at least 4 measurement occasions. Items were scored on a Likert scale ranging from 0 to 4. In items 1 and 4, higher scores indicate more stress (eg, …*how often have you felt that you were unable to control*...), but in items 2 and 3, higher scores indicate less stress (eg, …*how often have you felt confident about*...). Therefore, items 2 and 3 had to be inverted—(0=4) (1=3) (2=2) (3=1) (4=0)—to calculate the PSS-4 scale score so that higher scores indicate higher stress levels. The PSS-4 was repeatedly assessed on the mobile phone of TYS users. The implemented instruction was to rate the PSS-4 for the last week. The intraclass correlation (ICC) for the PSS-4 scale scores was rather high at ICC=0.70, suggesting a strong between-subject variance component. Using a multilevel confirmatory factor analysis framework [14], we estimated within- and between-subject reliability by calculating 2-level composite reliability (omega), which is appropriate for unit-weighted scoring of congeneric scales [15]. This resulted in *ω*_within_=0.60 (95% CI 0.51 to 0.68) and *ω*_between_=0.93 (95% CI 0.90 to 0.96).

#### Perceived Stress Reactivity Scale

The PSRS [5] was assessed once at the beginning of the study by each TYS user on the mobile phone. It measures stress reactivity, that is, interindividual differences in stress responses. The PSRS consists of 23 items, which were scored on a Likert scale ranging from 0 to 2. Higher scores indicate more perceived stress reactivity in some items (eg, *When tasks and duties build up to the extent that they are hard to manage*…), but less perceived stress reactivity in other items (eg, *When I want to relax after a hard day at work*…). Therefore, the following items had to be inverted—(0=2) (1=1) (2=0)—to calculate the perceived stress reactivity subscales and total scale so that higher scale scores indicate higher perceived stress reactivity: Items 2, 10, 20, 8, 13, 15, 18, 5, 17, 19, 11, 22. The subscales and their internal consistencies (Cronbach’s Alpha) in our sample were as follows: *prolonged reactivity* (alpha=.76), *reactivity to failure* (alpha=.63), *reactivity to social conflicts* (alpha=.74), *reactivity to work overload* (alpha=.86), and *reactivity to social evaluation* (alpha=.66). The total scale had an internal consistency (Cronbach alpha) of alpha=.87 in our sample.

#### TrackYourStress Mobile Health Crowdsensing Platform

TYS is an mHealth crowdsensing platform, which offers a website (registration and account management), an Android and iOS mobile app, a MariaDB relational database for the central repository to store the collected data, and a sophisticated Representational State Transfer (ie, RESTful) application program interface (API) for communication between the mobile apps, website, and database. A total of 4 questionnaire types, all related to stress, were implemented and integrated into TYS, namely registration, daily, weekly, and monthly questionnaire. In addition, the environmental sound level and the GPS position can be measured by the mobile apps, but TYS users must allow these sensor measurements when registering to TYS.

**Figure 1:**
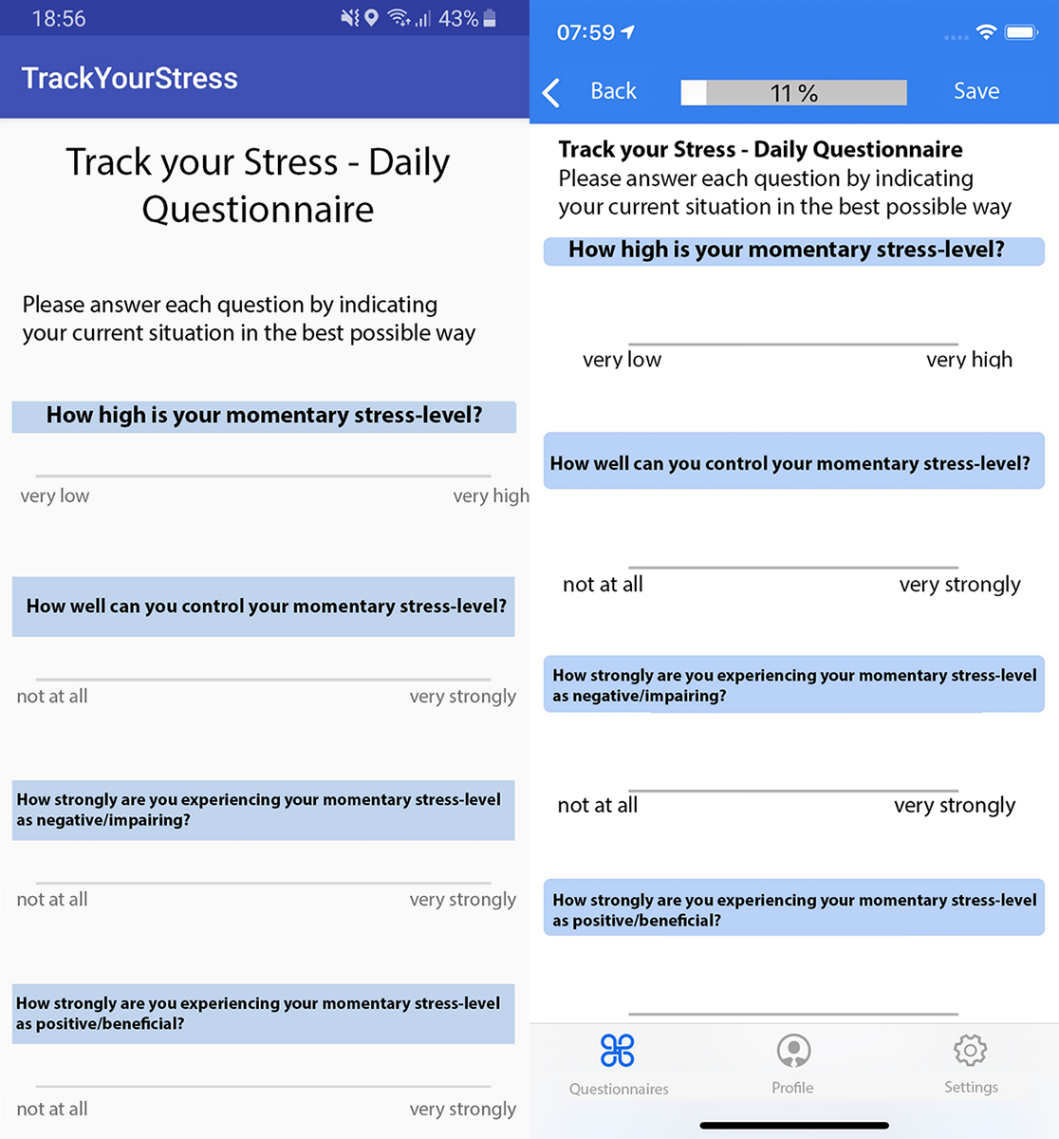
Impression of the daily assessment questionnaire (left Android; right iOS).

The applied procedure for all TYS users, in turn, is as follows: First, they register through the website or the mobile apps. Second, users have to fill in the registration questionnaire once. This includes demographic data (eg, gender and date of birth), the PSS-4, the PSRS, and the coping scales of the Stress and Coping Inventory (SCI; [16]). In a future version of TYS, a personality measure will be included in the registration questionnaire as well. Following the completion of the registration questionnaire the continuous mobile crowdsensing procedure starts, that is, filling out daily, weekly, and monthly questionnaires as well as automatically measuring the environmental sound level and GPS position. Note that the questions of the daily questionnaire can be obtained from Table 1. In particular, 3 basic user interface elements were implemented to answer a question by a TYS user. First, we implemented sliders, which represent the Visual Analogue Scales. Second, only for Question 5 *What stresses you at the moment?* we implemented a user interface element called Category, which, in turn, shows the following 4 categories to a TYS user: nothing, work-related matters, private matters, other. The user can then select all those categories, which are currently stressful for him or her. Third, we implemented a user interface element to provide the so-called Self-Assessment Manikins (SAM) [17] on Android and iOS. The SAM, in turn, are built on pictograms and used in psychology to measure emotions. To get a better impression of selected user interface elements, Figure 1 shows how the questionnaires are presented on the 2 mobile operating systems. Finally, note that the weekly questionnaire includes the PSS-4, whereas the monthly questionnaire includes the coping scales of the SCI.

**Table 1:** Items of the TrackYourStress daily questionnaire.

Number	Question	Scale
1	How high is your momentary stress level?	VAS^a^
2	How well can you control your momentary stress level?	VAS
3	How strongly are you experiencing your momentary stress level as negative/impairing?	VAS
4	How strongly are you experiencing your momentary stress level as positive/beneficial?	VAS
5	What stresses you at the moment?	C^b^
6	How is your mood right now?	SAM^c^
7	How is your arousal right now?	SAM
8	How important is the current situation for you personally?	VAS
9	How would you assess your ability to cope with the currently experienced situation?	VAS

^a^VAS: Visual Analogue Scale.

^b^C: categories.

^c^SAM: Self-Assessment Manikins [17].

**Figure 2:**
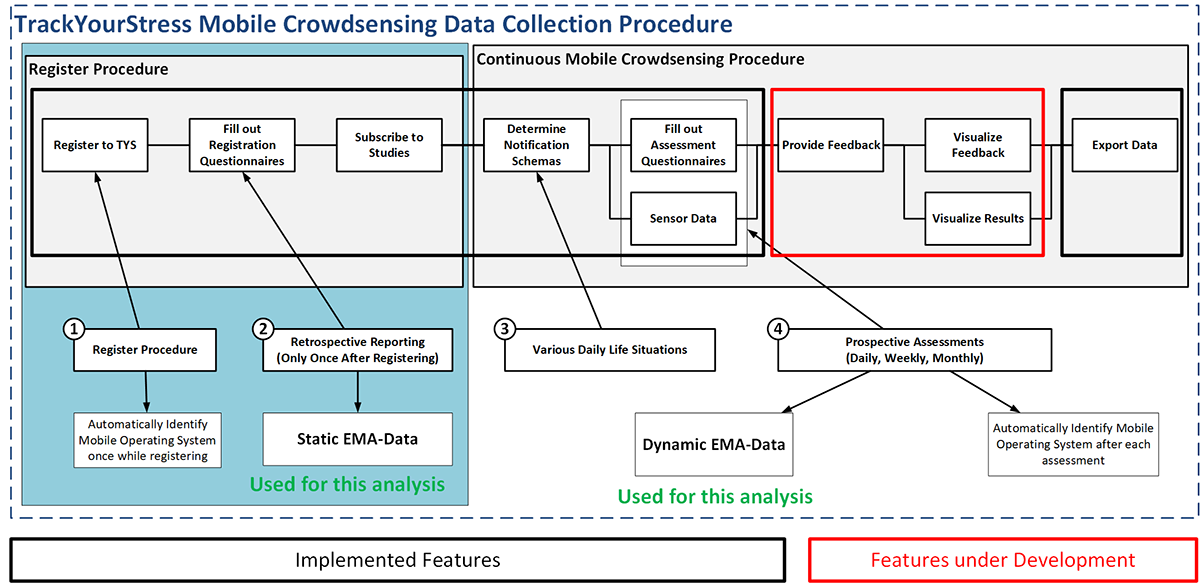
TrackYourStress mobile crowdsensing collection procedure.

For the crowdsensing procedure, users have to accept or select a predefined notification schema. It determines the frequency and in what way (ie, fixed or random points in time) the daily, weekly, and monthly questionnaires are applied. Each time a notification appears, the user may click on it to start the mobile app (if not already running), and the respective questionnaire (daily, weekly, or monthly) is then directly shown to the user. Then, he/she can fill in the questionnaire. It is also possible for users to fill in the questionnaires without notifications. While filling out the questionnaire, either with or without using a notification, the GPS position and the environmental sound level are measured (if the app is allowed to measure them). After completion, the results are transferred to the database through the RESTful API if the mobile app is on the Web, otherwise the results are locally stored until the device gets a Web-based connection. For a detailed technical description of these features see [18,19]. The website and the RESTful API of TYS are publicly released, whereas the smart mobile apps are not yet distributed through the official mobile app stores from Apple and Google. Therefore, we used a TestFlight-based distribution for iOS and downloadable Android packages for Android. Presently, TYS is only available in German; however, we are currently translating it to English. Figure 2 summarizes the data collection procedure for TYS with its implemented and planned features. Regarding TYS in general, 2 further important aspects are finally mentioned. First, the source code of TYS is currently not freely available but will be released to the research community in the near future (ie, after several aspects have been added for using it more conveniently; eg, by adding English as another possible language to the platform). Second, we plan to use TYS for several other purposes beyond this study. For example, it is planned to offer it to companies to anonymously track the stress levels of their employees over time. Furthermore, it is planned to give individuals the possibility to adjust the existing questionnaires to their individual needs. If they feel that other questions would fit better to their individual situation, then they should be able to adjust the daily, weekly, and monthly questionnaire. In addition, it is planned to integrate more sensors like the ones from Empatica (eg, [20]) to provide even more opportunities to the TYS users.

### Study Design

A total of 3 students of the FOM University of Applied Sciences in Augsburg and Munich (Germany) recruited participants. The 3 students asked their social networks (students, friends, family members, and colleagues) whether they are interested to partake in the study. Participants interested to partake first had to provide written informed consent before they got access to TYS. They downloaded the app and went through the TYS procedure as described earlier. The participants were informed that they should use TYS for at least 4 weeks in their daily life.

### Participants

After excluding test users, N=113 individuals used TYS for this study. The participants were asked to provide at least 5 PSS-4 assessments during the study interval, that is, filling in the PSS-4 in the TYS registration questionnaire and filling in at least 4 weekly TYS questionnaires in the upcoming weeks. One weekly TYS questionnaire less than intended was tolerated to address the RQs. Therefore, the inclusion criteria for this study were filling in the PSRS and the PSS-4 in the registration questionnaire and completing at least three weekly questionnaires including the PSS-4. We deleted all PSS-4 assessments given within an interassessment interval of 24 hours, as some users filled in the PSS-4 several times a day. This resulted in a sample of 78 participants for this study. The sample description is provided in Table 2 as is the comparison between included (n=78) and excluded study participants (n=35) in baseline variables (gender, age, PSS-4 at registration, and PSRS scales at registration). Included and excluded participants differed in age, with included participants being significantly older than excluded participants (*P*=.005). The corresponding effect size was medium (Hedges g=0.52). No significant differences emerged for gender, stress level at registration, and perceived stress reactivity at registration.

**Table 2:** Sample description and statistical comparisons between included and excluded participants in baseline variables.

Variable	Included sample (n=78)	Excluded sample (n=35)	Values	
			*t* (*df*)	*P* value
Female, n (%)	50 (64.1)	15 (44.1)	—^a^	.06^b^
Age (years), mean (SD)	35.04 (12.30)	28.98 (9.22)	−2.90 (85.82)	.005
PSS-4^c^, mean (SD)	5.32 (3.00)	5.17 (3.18)	−0.24 (111)	.81
PSRS^d^ prolonged reactivity, mean (SD)	2.81 (1.97)	2.94 (1.80)	0.35 (111)	.73
PSRS reactivity to failure, mean (SD)	4.73 (1.58)	4.54 (1.63)	−0.58 (111)	.57
PSRS reactivity to social conflicts, mean (SD)	6.32 (2.21)	5.69 (2.40)	−1.37 (111)	.17
PSRS reactivity to work overload, mean (SD)	3.60 (2.65)	3.06 (2.27)	−1.06 (111)	.29
PSRS reactivity to social evaluation, mean (SD)	4.59 (2.46)	4.06 (2.51)	−1.06 (111)	.29
PSRS total, mean (SD)	22.05 (7.69)	20.29 (8.29)	−1.10 (111)	.27

^a^Not applicable.

^b^Fisher exact test.

^c^PSS: Perceived Stress Scale.

^d^PSRS: Perceived Stress Reactivity Scale.

Our average PSS-4 baseline score of 5.32 is comparable with a previously reported mean of 5.43 based on a large sample of N=37,451 participants [11]. A *t* test using the means, standard deviations, and sample sizes showed that our PSS-4 mean is not significantly different (*t*_37,527_=−0.328; *P*=.74) from this previously reported one [11]. Our PSRS scores correspond to the ones previously depicted by Schlotz et al (see Figure 2 in their paper) [5] for a German sample between 26 and 60 years. We could not statistically compare the PSRS scores based on means, standard deviations, and sample sizes, as they are only graphically illustrated in the study by Schlotz et al [5].

### Statistics

IBM SPSS v25, Stata v15.1, and Mplus v7.3 were used for statistical analyses. All statistical tests were 2-tailed, and the significance level was set to *P*<.05. To address the RQs, linear multilevel models were used. Multilevel models account for the nested data structure (assessments nested within users), are flexible in handling missing data, do not require the same amount of data points per participants, and do not require equidistant measurement points [21-23]. All multilevel models were calculated with the full maximum likelihood estimation and had assessments as level 1 and participants as level 2. As random terms, the random intercept and the random slope were included in all models, that is, the random slope term makes sure that time is included as a random effect. The unstructured variance-covariance matrix was selected in all models.

To address the RQs, the time variable was coded as follows. The time a user filled in the PSS-4 for the first time (TYS registration) was coded as 0. The further PSS-4 assessments of a user were coded as the amount of days after his/her first PSS-4 assessment, for example, the time of a PSS-4 assessment provided 8 days after his/her first PSS-4 assessment was coded as 8 and the time of a PSS-4 assessment provided 15 days after his/her first PSS-4 assessment was coded as 15.

For RQ1 (*How is the time trend of stress levels when using TYS in the entire population being studied in a first explorative analysis?*), the change of PSS-4 over time was evaluated. Therefore, 1 linear multilevel model with the PSS-4 as the dependent variable was performed, which investigated the fixed effect of time (days).

For RQ2 (*Is there an interaction between the time trend of stress levels when using TYS and interindividual differences in perceived stress reactivity?*), the interaction between time (days) and perceived stress reactivity was in focus. Thereby, 2 linear multilevel models were used. In both models, the PSS-4 was the dependent variable and the fixed effect of time (days) was evaluated. In addition, in 1 multilevel model, the fixed effects of the 5 subscales of the PSRS (time-invariant covariates) and their interactions with time (days) were investigated. In the other multilevel model, the total scale of the PSRS (time-invariant covariate) and its interaction with time (days) were evaluated as fixed effects. In both models, z-standardized PSRS scale scores were used.

## Results

In total, the included n=78 participants, who completed at least four PSS-4 assessments, provided 380 PSS-4 assessments in total. On an average, they completed the PSS-4 for a mean 4.87 (SD 0.75) times. The average time interval between 2 consecutive PSS-4 assessments was mean 7.04 (SD 2.70) days. The average time interval between the first and the last PSS-4 assessment of a participant amounted to mean 29.23 (SD 6.77) days.

### Results for Research Question 1

Tables 3 and 4 show the result of the linear multilevel model exploring the time trend of stress levels for the entire population being studied. It can be seen that the stress levels did not change over time when using TYS, since the fixed effect time (days) did not reach statistical significance (*P*=.83).

**Table 3:** Fixed effects of the linear multilevel model evaluating the time trend of perceived stress -levels (PSS-4) while using TrackYourStress.

Fixed effects	Estimate	SE	Values	
			*t* test (*df*)	*P* value
Intercept	5.397	0.344	15.677 (78.026)	<.001
Time	−0.003	0.013	−0.211 (65.862)	.83

**Table 4:** Random effects of the linear multilevel model evaluating the time trend of perceived stress -levels (PSS-4) while using TrackYourStress.

Random effects	Estimate	SE	Values	
			Wald Z test	*P* value
Var(Residual)	2.247	0.218	10.326	<.001
Var(Intercept)	7.910	1.485	5.328	<.001
Cov(Intercept; time)	−0.064	0.043	−1.480	.14
Var(Time)	0.008	0.002	3.421	.001

### Results for Research Question 2

**Figure 3:**
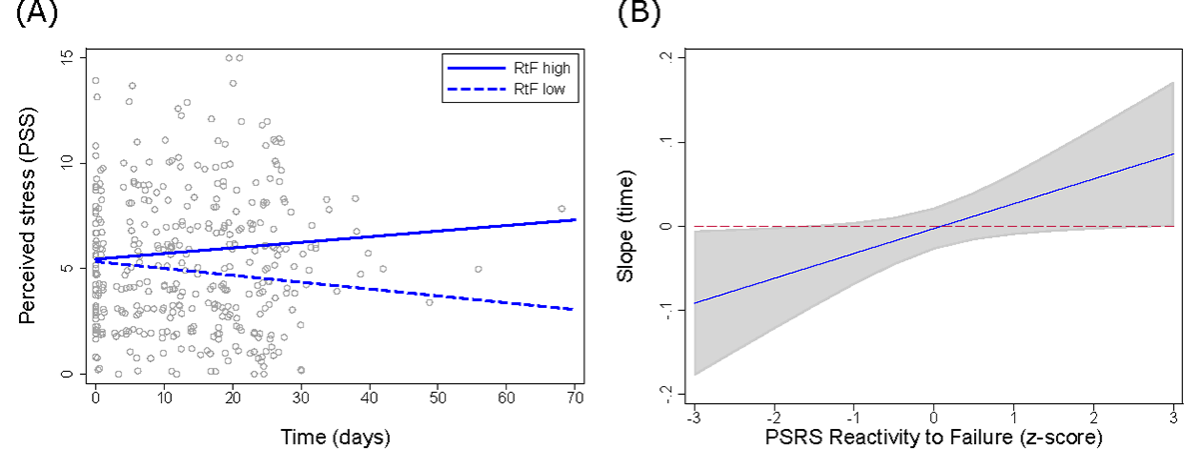
Illustration of the interaction between time (days) of perceived stress-levels (PSS-4) and the PSRS Reactivity to Failure (RtF) subscale. (A) Estimated simple slopes at 1 SD above and below mean RtF. (B) Margins and 95 % confidence interval for the time trend of stress-levels across a range of RtF scores.

Tables 5 and 6 present the results of the linear multilevel model testing interactions between the time trend of PSS-4 stress levels and the PSRS subscales. For the PSRS subscale *reactivity to failure*, the time x PSRS interaction was statistically significant (*P*=.04). Figure 3 illustrates the time trend of stress at different levels of the *reactivity to failure* subscale and a margin plot showing confidence intervals for the slope parameter at different levels (simple slopes). It can be seen that the higher the individual’s reactivity to failure, the more the stress levels increased over time while using TYS, although confidence intervals excluded zero only at relatively low and very high levels. Moreover, increases in the PSRS subscales *prolonged reactivity* (estimate=1.030; *P*=.002) and *reactivity to work overload* (estimate=0.895; *P*=.02) at baseline and registration respectively were associated with higher PSS-4 stress levels at baseline/registration.

Tables 7 and 8 show the results of the linear multilevel model that evaluated the interaction between the time trend of PSS-4 stress levels and the PSRS total scale. No significant interaction between changes of stress levels over time and the PSRS total scale emerged (*P*=.54). However, at baseline/registration, higher scores on the PSRS total scale were associated with higher PSS-4 stress levels (estimate=1.171; *P*<.001).

**Table 5:** Fixed effects of the linear multilevel model evaluating the time trend of perceived stress- levels (PSS-4) while using TrackYourStress including the 5 subscales of the Perceived Stress Reactivity Scale as z-standardized time-invariant covariates.

Fixed effects		Estimate	SE	Values	
				*t* test (*df*)	*P* value
**Intercept**		5.398	0.301	17.940 (78.800)	<.001
	PSRS^a^ prolonged reactivity	1.030	0.325	3.169 (79.275)	.002
	PSRS reactivity to failure	0.058	0.339	0.172 (78.743)	.86
	PSRS reactivity to social conflicts	−0.501	0.418	−1.198 (78.715)	.24
	PSRS reactivity to work overload	0.895	0.382	2.342 (78.177)	.02
	PSRS reactivity to social evaluation	0.393	0.368	1.069 (77.248)	.29
**Time**		−0.003	0.012	−0.238 (65.911)	.81
	Time × PSRS prolonged reactivity	−0.004	0.014	−0.292 (73.000)	.77
	Time × PSRS reactivity to failure	0.030	0.014	2.114 (64.951)	.04
	Time × PSRS reactivity to social conflicts	0.009	0.017	0.502 (66.230)	.62
	Time × PSRS reactivity to work overload	−0.014	0.016	−0.883 (63.165)	.38
	Time × PSRS reactivity to social evaluation	−0.005	0.015	−0.333 (63.636)	.74

^a^PSRS: Perceived Stress Reactivity Scale.

**Table 6:** Random effects of the linear multilevel model evaluating the time trend of perceived stress- levels (PSS-4) while using TrackYourStress including the 5 subscales of the Perceived Stress Reactivity Scale as z-standardized time-invariant covariates.

Random effects	Estimate	SE	Values	
			Wald Z test	*P* value
Var(Residual)	2.248	0.218	10.325	<.001
Var(Intercept)	5.731	1.131	5.069	<.001
Cov(Intercept; time)	−0.061	0.037	−1.651	.10
Var(Time)	0.007	0.002	3.260	.001

^a^PSRS: Perceived Stress Reactivity Scale.

**Table 7:** Fixed effects of the linear multilevel model evaluating the time trend of perceived stress- levels (PSS-4) while using TrackYourStress including the total scale of the Perceived Stress. Reactivity Scale as z-standardized time-invariant covariate.

Fixed effects	Estimate	SE	Values	
			*t* test (*df*)	*P* value
Intercept	5.393	0.318	16.958 (78.225)	<.001
PSRS total^a^	1.171	0.320	3.658 (78.317)	<.001
Time	−0.002	0.013	−0.186(65.547)	.85
Time × PSRS total	0.008	0.013	0.613 (68.266)	.54

^a^PSRS total: Perceived Stress Reactivity Scale total score.

**Table 8:** Random effects of the linear multilevel model evaluating the time trend of perceived stress- levels (PSS-4) while using TrackYourStress including the total scale of the Perceived Stress Reactivity Scale as z-standardized time-invariant covariate.

Random effects	Estimate	SE	Values	
			Wald Z test	*P* value
Var(Residual)	2.252	0.219	10.302	<.001
Var(Intercept)	6.553	1.267	5.172	<.001
Cov(Intercept; time)	-0.072	0.040	−1.797	.07
Var(Time)	0.008	0.002	3.382	.001

^a^PSRS total: Perceived Stress Reactivity Scale total score.

## Discussion

### Principal Findings

This nonclinical study evaluated the time trend of perceived stress levels while using the crowdsensing mHealth platform, TYS, and the influence of the participants’ perceived stress reactivity on longitudinally measured stress trajectories. In a first explorative analysis, we investigated the time trend of stress levels for the entire population being studied. We found no significant change of stress levels over time, and this null finding is in line with the clinical research indicating nonreactivity to EMAs [24-26]. Yet, these results should be interpreted with caution, as no change over time is the null hypothesis in statistical terms. A null hypothesis (here: no change of stress levels over time) cannot be accepted only because of a nonsignificant result; see, for example, work on equivalence and noninferiority testing [27,28]. Moreover, there are no standards on what a *true* null effect would be (eg, in terms of how narrow a confidence interval has to be), and the power of this study is far too low to test for a narrow confidence interval. In addition, we did not include a control condition and—even if there was no significant change in our study—the time trend of stress levels while using TYS could be significantly different from the time trend of stress levels in a control condition not using TYS.

Second, we analyzed whether interindividual differences in perceived stress reactivity influence the stress level trajectories while using TYS. As stress and stress reactivity were correlated in cross-sectional research [5], we wanted to explore whether the participants’ perceived stress reactivity at baseline is associated with longitudinally measured stress trajectories as well. In accordance with [5], we could replicate the cross-sectional correlation between the PSRS total scale and perceived stress levels at baseline, but there were no interactions between the participants’ perceived stress reactivity at baseline and the time trend of stress levels when using TYS except for one subscale of the PSRS. The higher the individual’s reactivity to failure, the more the stress levels increased while using TYS. More specifically, individuals with higher perceived stress reactivity to failure (eg, mistakes during work) reported an increase in stress levels over a 4-week period. Possibly, individuals with high perceived stress reactivity to failure are more aware of failures in daily routines when monitoring their stress levels in everyday life. Being more aware of stressors in daily life might help individuals with high perceived stress reactivity to adapt their stress responses in the long run [29]. Thus, linking TYS to ecological momentary interventions (EMIs) [30] such as mobile apps for training mindfulness might be a fruitful avenue for future stress research [31-34].

### Strengths and Limitations

Our research design does not allow inferring that it was specifically TYS that increased the stress levels in participants with higher reactivity to failure. There might be several confounders that influenced this result (eg, stressful life events and interpersonal problems with family or friends). This is related to the major limitation of this study, the rather low internal validity because of the lack of a control condition. A randomized controlled trial as for example planned by [35] should be performed with TYS in the future. A randomized controlled trial could compare TYS versus no TYS or specific TYS components with each other, as it could be that specific actions being executed with the TYS solution might be responsible for changes of stress levels.

Another limitation is that the reliability of the PSRS subscale *reactivity to failure* was rather low in this sample (alpha=.63). In the previous research, the Cronbach alpha values for the PSRS subscale *reactivity to failure* ranged between alpha=.65 and alpha=.73. This is likely because of the rather short scale comprising 4 items and might have biased our results. However, it should be noted that reliability estimates of that size are usually sufficient for group studies. Moreover, the external validity of the results is not very high as students of only one university recruited the sample, and the sample size was relatively small. To enhance the generalizability of the results, larger and more representative samples would be welcome. This could be realized by uploading TYS to the app stores. We plan to do this in the next months. Another threat to the external validity is the result that the TYS participants included in the current study were some years older than the TYS participants not meeting the inclusion criteria (mean 35.04 vs mean 28.98). The results might, therefore, be more representative for the TYS users being in their mid-thirties. Furthermore, the sample is rather heterogeneous, as the students recruiting the participants invited fellow students, friends, family members, colleagues, etc, to participate in the study. As we wanted to collect the data as anonymously as possible, the app did not ask about the relationship of the participants to the recruiting students (fellow student, friend, family member, and colleague). Therefore, we could not analyze how the participants’ relationship to the recruiting student might have influenced the results. As we cannot find out which of our users were students, we cannot analyze whether seasonality, part of term, or time of year influences the results. This needs to be addressed in future studies. Currently, we intend to perform a study comparing stress levels of students during exam periods with stress levels of students in periods without exams. Furthermore, TYS might be a helpful tool for companies to reduce psychological health problems at the workplace. For example, TYS might help to assess stressors at the workplace and support psychosocial risk assessment. Thus, we intend to build up a large TYS database and to provide personalized feedback to users about their stress levels in relation to their reference group with employees from different companies. Users scoring higher than the reference group could be guided to EMIs (see above), internet-based self-help programs, or to professionals offering face-to-face stress management interventions nearby. It also should be kept in mind that the psychometric properties of the PSS-4 have been criticized [9,10]. Our results regarding stress levels solely rely on the self-report PSS-4. Although between-subject reliability of the outcome variable’s scores was high, within-subject reliability was at a rather low level (*ω*_within_=0.60). This could be because of a number of factors such as the rather high ICC of 0.70, the relatively small clusters (ie, measurements per person), or the short scale (4 items). Although this reliability might be sufficient for group studies, future versions of the app should aim at increasing within-subject reliability of the outcome variable for achieving sufficient reliability of individual assessments. Results would be more robust when a self-report like the PSS-4 is combined with other stress assessment methods such as the Mobile Photographic Stress Meter [36] and objective measures, sensors, and computational methods [37] such as information gained from passive mobile phone sensing [38,39] or physiological signals [40,41]. Integrating such different measures of stress should be a next step in the development of TYS. Moreover, it should be investigated in a larger sample whether there are differences between iOS and Android users with regard to changes of stress levels while using TYS. Previous studies have shown that iOS and Android users differ from each other and this might confound results of mobile phone-based studies [42-44].

### Conclusions

In summary, this study suggested that TYS does not change perceived stress levels in general but that TYS might influence that stress levels increase in individuals with higher reactivity to failure. These results need to be replicated in studies with a control condition and a larger more representative sample.

## Abbreviations

API: application program interface
EMA: ecological momentary assessment
EMI: ecological momentary intervention
GPS: Global Positioning System
ICC: intraclass correlation
PSRS: Perceived Stress Reactivity Scale
PSS: Perceived Stress Scale
RESTful: Representational State Transfer
RQ: research question
SAM: Self-Assessment Manikins
TYS: TrackYourStress
